# MYC is in the CARDs: CBM complexes coordinate immune and MYC-dependent cellular function

**DOI:** 10.3389/fmmed.2025.1731823

**Published:** 2025-11-19

**Authors:** Stanley B. DeVore, Gurjit K. Khurana Hershey

**Affiliations:** 1 University of Cincinnati College of Medicine, Cincinnati, OH, United States; 2 Division of Asthma Research, Cincinnati Children’s Hospital Medical Center, Cincinnati, OH, United States

**Keywords:** Myc (c-Myc), CARD11 (CARMA1), CARD14 (CARMA2), CARD9, MALT1, CBM complex, NF-kappaB (NF-κB), CARD10 (CARMA3)

## Abstract

MYC is a transcription factor crucial for a host of cellular functions from proliferation to metabolism, and MYC dysregulation contributes to disease pathogenesis. A growing body of evidence suggests that MYC signaling is regulated by the caspase activation and recruitment domain-coiled-coil (CARD-CC) proteins: a family of immunological signaling mediators that canonically drive NF-κB signaling across nearly all tissues. MYC regulation coordinated by the CARD-CC proteins occurs by multiple mechanisms, including transcription, physical binding, and subcellular localization. Herein, we highlight the hallmark studies that collectively broaden the sphere of influence of CBM complexes beyond NF-κB to include MYC, which has functional impact on cells within and likely beyond the immune system. The studies reviewed herein provide rationale for future studies that examine non-canonical CBM-MYC signaling, its relationship with canonical NF-κB signaling, and its contribution to human health and disease.

## Introduction

MYC, or c-Myc, is a critical transcription factor that, when appropriately regulated, regulates physiological processes ranging from proliferation and metabolism to differentiation and apoptosis in virtually all tissues (Jha et al., 2023). Precise regulation of MYC is crucial, however, as its dysregulation mediates a wide range of diseases (Jha et al., 2023). Separately, the caspase activation and recruitment domain-coiled-coil (CARD-CC) family of proteins are important signaling mediators within the innate and adaptive immune systems in particular (DeVore and Hershey, 2022). These proteins form signalosomes with B-cell lymphoma/leukemia 10 (BCL10) and mucosa-associated lymphoid tissue lymphoma translocation protein 1 (MALT1) known as “CBM complexes,” which canonically drive pro-inflammatory NF-κB signaling. To date, the literature on CBM complexes has largely focused on this NF-κB signaling; however, there is emerging evidence that these signalosomes are novel regulators of MYC within and beyond the immune system, which we will discuss in the following sections. After introducing MYC and the CARD-CC family, we will review the published literature supporting the regulatory roles of CARD11 and CARD14 in MYC signaling and how this influences tissue homeostasis and disease. Though no studies have yet directly confirmed CARD9-MYC and CARD10-MYC signaling, we will also speculate on these potential pathways based on available studies. Further, we will highlight future research directions that may impact how the scientific community approaches the study and treatment of CBM- or MYC-dependent diseases.

## MYC in cellular function and disease

Mammalian cells rely on the complex coordination of numerous signaling pathways to regulate cellular function, and the ubiquitous transcription factor MYC, encoded by *MYC,* is central to many of them. Though MYC can canonically drive target gene expression by directly binding DNA, it is also considered to be a global amplifier of all active genes because of its influence on both transcriptional activators and transcriptional machinery (Jha et al., 2023). As such, MYC exerts significant control over cellular processes crucial for tissue homeostasis, including proliferation, differentiation, and cellular survival.

Within the immune system, MYC directs the balance between self-renewal and differentiation in hematopoietic stem cells, mediates the development of B- and T-cells, and sustains myeloid populations (Ahmadi et al., 2021; Delgado and León, 2010). MYC is also crucial for maintaining the epithelial barriers that serve as the body’s first line of defense against environmental threats (e.g., allergens, microbes) by regulating self-renewal of basal keratinocytes and initiating terminal differentiation of suprabasal keratinocytes into the stratum corneum (DeVore et al., 2024).

The fundamental role of MYC is highlighted by the disease states resulting from its dysregulation (Zacarías-Fluck et al., 2024). Unchecked MYC signaling is oncogenic by orchestrating uncontrolled proliferation and cellular metabolism, impeding tumor-suppressive mechanisms and promoting genomic instability (Dhanasekaran et al., 2022). Several hematological malignancies are driven by or associated with MYC dysregulation (Delgado and León, 2010), including neoplasms of B-cells, T-cells, plasma cells, and myeloid lineages (Delgado and León, 2010). MYC dysregulation is also associated with epithelial malignancies, including cutaneous squamous cell carcinomas and their precursor actinic keratoses (Hameetman et al., 2013; Kim et al., 2022; Toll et al., 2009) as well as carcinomas of the head and neck, lung, esophagus, and colon (Kalkat et al., 2017).

Since even modest changes in MYC protein levels can alter cellular responses or drive oncogenesis (Dhanasekaran et al., 2022; Liu et al., 2023), MYC is subject to multiple levels of regulation which are comprehensively reviewed elsewhere (Jha et al., 2023; Lee et al., 2023). Briefly, *MYC* gene transcription is controlled by multiple promoters and initiation sites and influenced cis-regulatory elements, DNA conformational changes, and other transcription factors (e.g., SP1, TGFβ, p53). *MYC* transcript stability and translation are modulated by post-transcriptional modification, RNA-binding proteins and micro-RNAs. At the protein level, subcellular localization, binding partners, and post-translational modifications (PTMs; e.g., phosphorylation, acetylation, and ubiquitination) influence MYC stability and transcriptional activity (Lee et al., 2023). Finally, MYC is canonically downregulated by proteasomal degradation (Jha et al., 2023). However, the number of recognized MYC regulatory mechanisms continues to grow. As discussed below, our group recently demonstrated that MYC can be degraded by selective autophagy in keratinocytes (DeVore et al., 2024)—a mechanism since noted in colorectal cancer (Ye et al., 2024). Further, MYC in myeloid cells is subjected to K63-linked polyubiquitination by TRAF6, which counters an activating acetylation PTM to prevent leukemogenesis (Jha et al., 2023; Muto et al., 2022). The elucidation and study of MYC regulatory mechanisms are key to future interventions targeting MYC signaling for the prevention or treatment of MYC-associated diseases.

## The CARD-CC-BCL10-MALT1 (CBM) complex

Upon detection of an environmental threat, the innate and adaptive immune systems synergistically mount a protective immune response. This outcome is dependent on multiple families of pro-inflammatory signaling complexes that are activated in response to upstream triggers, such as pattern recognition receptors ligated by pathogen-associated molecular patterns. Upon activation, specific proteins nucleate the assembly of large supramolecular complexes that amplify and modulate signal transduction, which is necessary for immune effector functions. One notable signalosome is the Myddosome: in response to upstream toll-like receptors (TLRs), the Myddosome is nucleated by myeloid differentiation factor 88 (MyD88) and signals through TRAF6 to induce NF-κB and other pathways that induce cytokine and chemokine expression (Balka and De Nardo, 2019). Numerous other signalosomes, including inflammasomes (Broz and Dixit, 2016), have been described and are reviewed elsewhere (Xia et al., 2021).

**FIGURE 1:**
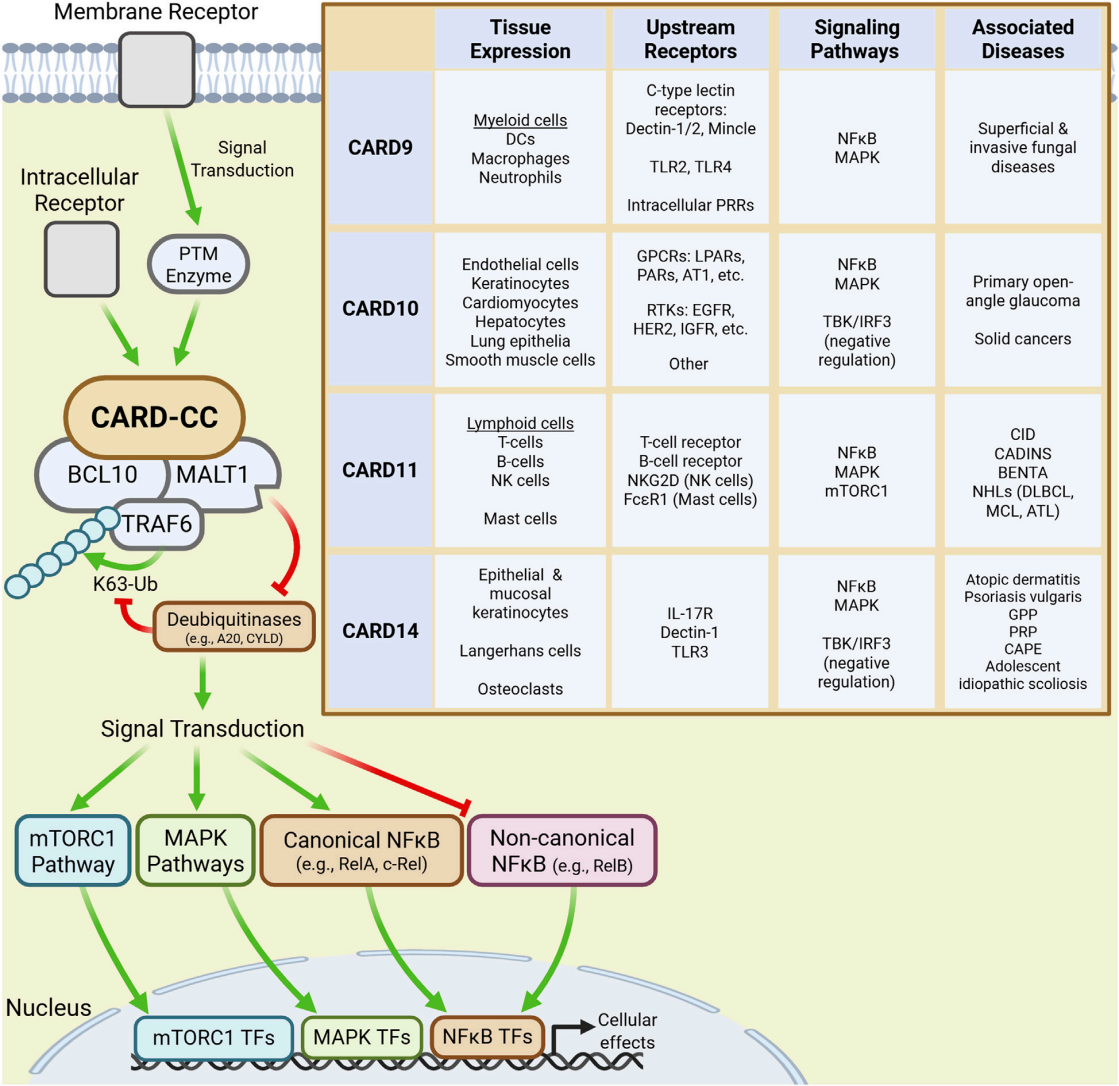
CARD-CC signaling and characteristics. Signal transduction downstream of membrane and intracellular receptors activate CARD-CC paralogs leading to the recruitment of BCL10 and MALT1 into a CBM complex. Recruited TRAF6 then K63-polyubiquitinates CBM components which scaffold modulators of downstream signaling pathways leading to changes in cellular function. The inset table summarizes the tissue expression profile, upstream receptors, signaling pathways and major disease associations for each CARD-CC paralog.

CARD-CC-BCL10-MALT1 (CBM) signalosomes are one family of immunological signaling complexes that have gained significant attention because of their roles in T-cell receptor (TCR) and B-cell receptor (BCR) signal transduction. CBM complexes are nucleated by the four paralogs of the CARD-CC family: CARD9, CARD10, CARD11, and CARD14. Though their expression is particularly prominent within the immune system, the CARD-CC paralogs are collectively expressed in nearly all tissues. Broadly, CARD9 is expressed in myeloid cells, CARD10 in endothelia and solid organs, CARD11 in lymphoid cells, and CARD14 in epidermal and mucosal epithelial cells (Figure 1) (DeVore and Hershey, 2022).

Inactive CARD-CC paralogs are maintained in an autoinhibited conformation by an inhibitor domain. Upstream ligand-receptor pairs, which differ by paralog and cellular context, activate CARD-CC proteins by mediating PTMs (e.g., phosphorylation) of inhibitory domain residues (Bedsaul et al., 2018). Notably, many disease-associated gain-of-function (GoF) mutations affect residues in autoinhibitory regions, thereby bypassing PTM-dependent activation (Bedsaul et al., 2018; Mellett, 2020). Canonically, each activated paralog recruits BCL10 and MALT1 into a supramolecular CBM complex which, like the Myddosome, uses TRAF6-mediated K63-linked polyubiquitination to activate NF-κB signaling. The protease activity of MALT1 potentiates this signaling by cleaving deubiquitinases that would otherwise disassemble the polyubiquitin chains (DeVore and Hershey, 2022; Bedsaul et al., 2018). Ultimately, CBM signaling drives the expression of pro-inflammatory cytokines, chemokines, and even antimicrobial peptides (AMPs) that offer primary protection and modulate downstream immune responses (DeVore and Hershey, 2022).

However, CBM signalosomes also influence immunological pathways other than the canonical NF-κB pathway (DeVore and Hershey, 2022; Mellett, 2020; Mei et al., 2016), such as the non-canonical NF-κB and antiviral TBK/IRF3 pathways. CBMs also signal through pathways that influence cellular growth, proliferation, differentiation and metabolism, including the mitogen-activated protein kinase (MAPK) and mTORC1 pathways (DeVore and Hershey, 2022). Further, MALT1 paracaspase activity modulates cellular activity by cleaving proteins that affect NF-κB and MAPK signaling transduction, RNA transcript stability (regnase-1, roquin-1/2), cytoskeletal regulation and cell growth (LIM domain and actin-binding protein 1) and even components of the CBM itself (Ruland and Hartjes, 2019).

Only recently has it become evident that CBM signalosomes can regulate MYC, which expands the sphere of influence of the CBM to include MYC-driven processes central to the immune system. The evidence supporting CBM-MYC signaling downstream of each paralog is discussed in the following sections.

## CARD11-MYC signaling

CARD11 (CARMA1) is expressed in lymphoid cells including T-cells, B-cells, natural killer cells; and in mast cells. CARD11 is the most well-understood paralog given its crucial roles in BCR and TCR signal transduction: it is essential for the activation, proliferation, and survival of lymphocytes and for the differentiation of specific effector T-cell (T_eff_) subsets including regulatory T-cells (T_reg_), T follicular helper cells, and T-helper 17 (T_H_17) cells (Lee et al., 2017; Brüstle et al., 2017; Molinero et al., 2009; Molinero et al., 2012; Egawa et al., 2003; Barnes et al., 2009). Germline absence of CARD11 causes combined immunodeficiency (CID), whereas germline hypomorphic and GoF mutations drive the immune dysregulation syndromes CARD11-associated atopy with dominant interference of NF-κB signaling (CADINS) and B-cell expansion with NF-κB and T-cell anergy (BENTA), respectively (DeVore and Hershey, 2022). The significant influence of CARD11 signaling in lymphocytes is also demonstrated by its role in hematologic malignancies: GoF mutations and activating gene fusions (i.e., *CARD11-PIK3R3*) are associated with hematologic malignancies including B-cell and T-cell non-Hodgkin lymphomas (Ruland and Hartjes, 2019; Garcia et al., 2024).

**FIGURE 2:**
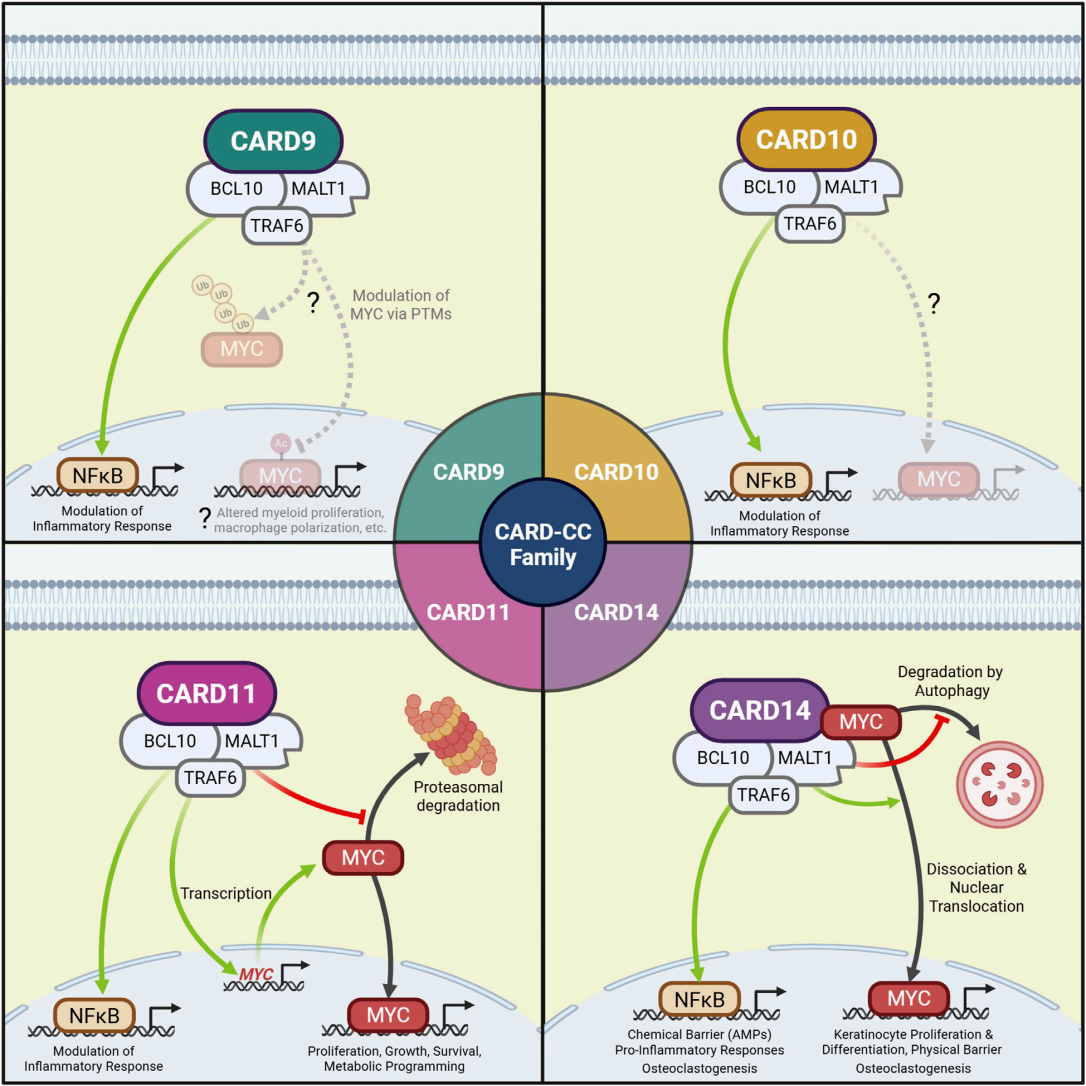
CBM complexes may regulate MYC at multiple levels to coordinate cellular function. CARD9: CARD9-MYC signaling has yet to be confirmed but may include altered post-translational modification of MYC. CARD10: CARD10-MYC signaling has yet to be directly observed. CARD11: The CARD11 CBM promotes *MYC* gene transcription in T_reg_ cells and prevents MYC proteasomal degradation in B-cells, resulting in altered proliferation, growth, survival and metabolic programming. CARD14: The CARD14 CBM directly binds MYC, which dissociates upon CARD14 activation to mediate transcriptional activity in the nucleus. CARD14 and MALT1 signaling also prevent MYC autophagic degradation. These events lead to keratinocyte proliferation, differentiation, and physical barrier formation, which complement the immunomodulatory effects of CARD14-NF-κB. In bone marrow-derived macrophages, CARD14-MYC and CARD14-NF-κB signaling together drive osteoclastogenesis.

MYC regulation by the CBM signalosome was first noted in B-cell lymphoma, where CARD11 can drive MYC activity to promote cellular proliferation (Figure 2). In mantle cell lymphoma (MCL) BCR-CARD11-MALT1 signaling supports MYC activity by reducing its proteasomal degradation. Further, proliferation and survival of MCL cell lines are MYC-dependent, demonstrating that CARD11-MYC is a crucial pathway for sustaining MCL (Dai et al., 2017). CARD11-MYC signaling also occurs in untransformed B-cells: MYC levels are reduced in splenocytes with catalytically-inactive MALT1 (Dai et al., 2017). However, in a separate study on diffuse large B-cell lymphoma, GoF CARD11 mutants behaved as a “second-hit” to constitutively-expressed MYC in lymphomagenesis, suggesting CARD11 and MYC participate in independent but complementary pathways (Reimann et al., 2021). However, CARD11-MYC signaling was not directly tested.

The CARD11 CBM is important for thymic T_reg_ development (Molinero et al., 2009; Barnes et al., 2009). While human versus mice phenotypes differ, deficient or impaired CARD11 or MALT1 signaling can cause systemic inflammatory disease possibly due to greater impairment of T_reg_ cells than T_eff_ cells (Carter and Pomerantz, 2022; Dorjbal et al., 2019; Gewies et al., 2014; Bornancin et al., 2015; Charbit-Henrion et al., 2017; Ma et al., 2017; Jaworski et al., 2014). NF-κB is important in T_reg_ development (Oh et al., 2017); however, T_reg_ defects associated with CARD11 deficiency are not fully complemented by NF-κB rescue (Lee et al., 2010), suggesting involvement of other downstream pathways. Notably, CARD11-MYC signaling has recently been demonstrated in T_regs_. Rosenbaum *et al.* demonstrated that CARD11-MYC signaling induces *Myc* mRNA expression in T_reg_ cells (Rosenbaum et al., 2022). Notably, this mechanism enhances T_reg_ proliferation and the mitochondrial metabolism that is preferentially utilized by T_reg_ cells (Rosenbaum et al., 2022; Newton et al., 2016). Unlike B-cells, however, CBM signaling does not affect MYC proteasomal degradation (Rosenbaum et al., 2022). MALT1-MYC signaling also protects against fatal autoimmune disease (Rosenbaum et al., 2022). Interestingly, mice with protease-dead *MALT1* are phenotypically similar to mice with T_reg_-specific deficiency in MYC (Saravia et al., 2020). These data support that CARD11-MYC signaling may indeed play a separate but cooperative role to CARD11-NF-κB signaling in T_reg_ biology.

Like T_reg_ cells, T_eff_ cells are also dependent on both MYC and CARD11: MYC is required for T_eff_ proliferation, growth and metabolic reprogramming upon activation (Wang et al., 2011), while CARD11 is required for T_eff_ cytokine expression and proliferation. T_eff_ proliferation is even sensitive to disease-associated hypomorphic or GoF CARD11 mutations (Egawa et al., 2003; Dorjbal et al., 2019; Snow et al., 2012). However, in the same study by Rosenbaum et al., MALT1 paracaspase activity did not appear to influence *MYC* mRNA levels or mitochondrial metabolism in conventional CD4^+^ T-cells (Rosenbaum et al., 2022). Beyond this, no other studies have directly investigated CARD11-MYC signaling in T_eff_ cells. Future studies should consider other possible mechanisms by which CARD11 could regulate MYC to influence T_eff_ function, including by altering its proteasomal degradation (as in B-cells), or influencing its physical binding or autophagic degradation (as CARD14 does in keratinocytes; see next section). Such studies would also help determine whether CARD11-MYC signaling could contribute to T-cell malignancies such as adult T-cell leukemia/lymphoma, which harbors CARD11 GoF mutations in about 25% of cases (Kataoka et al., 2015) and can demonstrate increased MYC activity (Kameda et al., 2022; Yamagishi et al., 2021).

## CARD14-MYC signaling

CARD14 (CARMA2) is expressed in epidermal keratinocytes, mucosal epithelia and osteoclasts (DeVore et al., 2024; Luo et al., 2025). It is associated with inflammatory skin diseases, including atopic dermatitis (AD), psoriasis vulgaris (PsV), and rarer psoriatic entities like generalized pustular psoriasis (GPP), pityriasis rubra pilaris (PRP), and CARD14-associated papulosquamous eruption (CAPE) (DeVore et al., 2024). AD is associated with hypomorphic CARD14 variants, while the psoriatic diseases are associated with CARD14 GoF variants (DeVore et al., 2024; Mellett, 2020; DeVore et al., 2021; Peled et al., 2019; Craiglow et al., 2018). Functionally, CARD14 stimulates the expression of cytokines, chemokines, AMPs and crucial barrier genes like *FLG* (encoding filaggrin) (DeVore and Hershey, 2022) that are collectively important for a protective skin barrier.

CARD14-MYC signaling in keratinocytes was recently identified as a determinant of skin barrier integrity (Figure 2). After finding an association between the CARD14 R820W (rs11652075) variant and reduced epidermal *FLG* expression in children with AD (DeVore et al., 2021), our group noted prominently decreased MYC transcriptional signatures in keratinocytes with the variant despite only subtle decreases in NF-κB signaling (DeVore et al., 2024). MYC was similarly attenuated by MALT1 inhibition. Though CARD14 did not affect *MYC* expression or proteasomal degradation, we found CARD14 physically interacted with MYC in a manner dependent on CARD14 variants and activation status. Wild-type CARD14 activation promoted MYC dissociation, nuclear localization and transcriptional activity; however, the R820W variant and MALT1 inhibition both attenuated dissociation from the CBM and promoted MYC autophagic degradation (DeVore et al., 2024). Additional studies are required to explore the underlying mechanisms, such as testing whether MYC is purposefully targeted to the autophagosome (i.e., the CBM triggers its ubiquitination by TRAF6) (Ye et al., 2024; Muto et al., 2022) or if the undissociated MYC is collaterally degraded in complex with the CBM (which can by downregulated by autophagy) (DeVore and Hershey, 2022; Paul et al., 2012; Yang et al., 2012; Moreno-García et al., 2013). Functionally, dysregulated CARD14-MYC signaling impaired keratinocyte proliferation, keratinocyte differentiation (e.g., reduced *FLG*), and barrier function (DeVore and Hershey, 2022; DeVore et al., 2024). Elevated MYC signaling is also implicated in psoriasis (Elder et al., 1990; Leng et al., 2024), and skin expressing psoriasis-associated GoF CARD14 mutants have both elevated MYC signatures and psoriatic features (e.g., epidermal hyperproliferation, parakeratosis) (DeVore et al., 2024). These findings indicate that CARD14 promotes physical barrier immunity through MYC that complements chemical barrier immunity through NF-κB. Interestingly, some CARD14 mutations impact CARD14-NF-κB signaling independent of CARD14-MYC signaling (DeVore et al., 2024), which may explain the pleiotropic presentations of CARD14-associated skin disease.

Most recently, CARD14 has been shown to promote differentiation of bone marrow-derived macrophages into osteoclasts in a MYC-dependent fashion (Luo et al., 2025). Like keratinocytes, CARD14 orchestrates this by directly binding MYC, enhancing its stability, and promoting its nuclear translation. CARD14 concomitantly promotes NF-κB and MAPK signaling which are also crucial for osteoclastogenesis (Luo et al., 2025), again demonstrating cooperation between multiple CARD14-dependent pathways.

## CARD9-MYC signaling

CARD9 is predominantly expressed in myeloid cells and is crucial for antifungal immunity. In antigen presenting cells, receptors sensing fungal carbohydrates stimulate CARD9 which mediates the secretion of cytokines (e.g., IL-12, IL-23, IL-1β) that polarize T-cells towards antifungal T_H_1/T_H_17 immunophenotypes (Liu et al., 2022). Indeed, patients with deleterious CARD9 mutations often develop fungal infections and allergic inflammation attributed to abrogated Th17 and Th1 polarization, respectively (DeVore and Hershey, 2022; Ruland, 2008). Though no studies have yet definitively linked CARD9 to MYC regulation, there are data suggesting that CARD9 not only regulates MYC but that, in contrast to CARD11 and CARD14, it uniquely suppresses MYC signaling.

CARD9 is crucial for polarization of resting M0 macrophages to pro-inflammatory M1 macrophages and mediating their effector function downstream of inflammatory stimuli (Campuzano et al., 2020). MYC, however, is suppressed in M1 macrophages (Liu et al., 2016; Pello, 2016; Pello et al., 2012). In contrast, CARD9-deficiency promotes polarization towards anti-inflammatory M2 macrophages (Campuzano et al., 2020), which require MYC for activation and for which MYC is a cellular marker (Pello et al., 2012). These observations suggest antagonistic regulation of CARD9 on MYC in macrophages: increased CARD9 signaling may suppress MYC to drive M1 polarization, while reduced CARD9 signaling may disinhibit MYC to permit M2 polarization. However, specific studies are needed to explore this hypothesis.

Dysregulated MYC in myeloid progenitors can mediate progression to AML (Huang et al., 2006; Laurenti et al., 2008). In mice, myeloid TLR2 signaling through MyD88 and TRAF6 suppresses MYC and subsequent progression to AML (Muto et al., 2022). Notably, TLR2-MyD88 signaling can also activate the CARD9 CBM complex (Wang et al., 2020; Hara et al., 2007; Dong et al., 2006), which also employs TRAF6 (Gorjestani et al., 2012). It would be interesting to test if CARD9 is either contributory or necessary for TRAF6-dependent MYC downregulation.

## CARD10-MYC signaling

The fourth CARD-CC paralog is CARD10 (CARMA3), whose expression is more variable and includes endothelial cells, smooth muscle cells, epidermal and respiratory epithelia, and the mesenchyme of several solid organs. CARD10 mutations are associated with primary open-angle glaucoma and are implicated in various neoplasms (e.g., bladder and breast) (Zhou et al., 2016; Ekambaram et al., 2018; Man et al., 2019). CARD10 CBM signaling is primarily pro-inflammatory, and is the only paralog known to be activated by receptor tyrosine kinase and G-protein coupled receptors (DeVore and Hershey, 2022; Ruland and Hartjes, 2019).

No focused studies have directly investigated a CARD10-MYC signaling pathway. However, many cancer-associated CARD10 mutations do not influence NF-κB signaling (Staal et al., 2024), suggesting other pathways—possibly including MYC—may be affected instead. In one study, CARD10 knockdown reduced β-catenin-mediated MYC expression and attenuated the invasion and proliferation of bladder carcinoma cells. β-catenin overexpression, however, rescued MYC and cellular invasion and proliferation, suggesting there may be a CARD10-β-catenin-MYC signaling axis (Man et al., 2019). Further, CARD10 is activated by the GPCRs lysophosphatidic acid receptor 2 and angiotensin II type 1 receptor which can also activate MYC (Ekambaram et al., 2018; Taghavi et al., 2008; Sun, 2010)—two other contexts in which CARD10-MYC signaling may be possible. However, additional studies are required to robustly test for CARD10-MYC signaling in CARD10-expressing tissues and how this may contribute to CARD10-associated disease.

## Conclusion and outlook

The elucidation of novel signaling pathways is crucial for not only understanding homeostatic function of cellular systems, but also for identifying previously unrecognized disease mechanisms that could be targeted therapeutically. Given both the widespread expression of CBM components and the significant cellular influence and pathogenic potential of MYC, the emerging evidence supporting CBM-MYC regulation is worth highlighting.

CBM complexes have been noted to regulate MYC by not only affecting its transcription but also by influencing its subcellular localization and protein stability (Figure 2). We believe it is likely that CBM complexes affect MYC by other mechanisms as well, including transcript stability, translation, or even post-translational modification (such as by TRAF6-mediated ubiquitination). Further, these regulatory mechanisms appear to be cell-type and/or paralog-specific, exemplified by the effect of CARD11 on MYC proteasomal degradation in B-cells but not T_reg_ cells, and the influence of CARD11 (but not CARD14) on *MYC* transcription. Future studies centered on the CBM should thus not only consider CBM-dependent MYC signaling but also consider the various levels of MYC regulation it may influence.

While CARD11 and CARD14 promote MYC signaling, early data suggest CARD9 may downregulate MYC. This could be explained by lack of the protein-binding membrane-associated guanylate kinase (MAGUK) domain in CARD9 (DeVore and Hershey, 2022), which is predicted *in silico* to bind MYC (DeVore et al., 2024). However, future studies are required to investigate whether the MAGUK domain is needed to promote MYC signaling and if its absence could permit MYC suppression by CARD9 or even the MAGUK-deficient splice-variant of CARD14 (CARD14*sh*).

Finally, CBM-dependent MYC signaling appears to be a functionally complementary to canonical CBM-NF-κB signaling, which may explain the pleiotropy of CBM-associated disease: the development or proliferation of B-cells and T_reg_ cells is partially dependent on both CARD11-MYC and CARD11-NF-κB signaling; the physical skin barrier induced by CARD14-MYC signaling complements the chemical barrier (e.g., AMP and cytokine expression) driven by CARD14-NF-κB; and both CARD14-MYC and CARD14-NF-κB signaling promote osteoclastogenesis. Additional studies are needed to define the roles of CBM signaling through MYC versus NF-κB (or other pathways) and how they interact. This work would contribute to a broader understanding of how the CBM controls cellular function and may elucidate novel approaches to treating associated pathologies.

## Data Availability

The original contributions presented in the study are included in the article/supplementary material, further inquiries can be directed to the corresponding author.

## Abbreviations

AD, Atopic dermatitis; AML, Acute myeloid leukemia; AMP, Antimicrobial peptide; BCL10, B-cell lymphoma/leukemia 10; BCR, B-cell receptor; BENTA, B-cell expansion with NF-κB and T-cell anergy; CADINS, CARD11-associated atopy with dominant interference of NF-κB signaling; CAPE, CARD14-associated papulosquamous eruption; CARD-CC, Caspase activation and recruitment domain coiled-coil; CBM complex, CARD-CC-BCL10-MALT1 complex; CID, Combined immunodeficiency; FLG, Filaggrin; GPP, Generalized pustular psoriasis; MAGUK, Membrane-associated guanylate kinase; MALT, Mucosa-associated lymphoid tissue lymphoma translocation protein 1; MAPK, Mitogen-activated protein kinase; MCL, Mantle cell lymphoma; PAR, Protease-activated receptor; PTM, Post-translational modification; TCR, T-cell receptor; TGFβ, Tumor growth factor β; T_eff_ cell, Effector T-cell; T_H_ cell, T-helper cell; T_reg_ cell, Regulatory T-cell; TLR, Toll-like receptor; TNFα, Tumor necrosis factor α.
